# The Fitness, Rest, and Exercise for Strength and Health (FRESH) Study: A Three-Year Comparison of College Students’ Perceived and Measured Health Metrics

**DOI:** 10.3390/nu17020217

**Published:** 2025-01-08

**Authors:** Julia Blouin, Adelaide Feek, Yichen Jin, Jennifer Cook, Timothy O’Neal, Jennifer M. Sacheck

**Affiliations:** Department of Exercise and Nutrition Sciences, Milken Institute School of Public Health, The George Washington University, Washington, DC 20052, USAyjin@gwu.edu (Y.J.); timothy.oneal@gwu.edu (T.O.); jsacheck25@gwu.edu (J.M.S.)

**Keywords:** college health, dietary patterns, food insecurity, mental health, stress, physical activity

## Abstract

The undergraduate college years are a critical transition period for young adults in establishing life-long health behaviors. **Background/Objective**: Within the FRESH Study, we aimed to understand the relationship between perceived physical health, perceived mental health, and specific health metrics (e.g., physical activity, food insecurity, sleep quality) among college students following the COVID-19 pandemic. **Methods**: First-year undergraduate students (*n* = 271) from an urban university were recruited in three study waves (2021–2022, 2022–2023, and 2023–2024). Participants answered online surveys on demographics, health perceptions, physical activity, dietary patterns, beverage consumption, food insecurity, stress, and sleep quality. **Results**: Overall, participants rated their physical health better than their mental health (‘good’, ‘very good’, or ‘excellent’; 89.6% vs. 62.2%), even though 30.7% were not of ‘healthy weight’ status, 34.5% did not meet physical activity recommendations, and 42.2% of students consumed some sugar-sweetened beverages (SSBs). Students reporting suboptimal (‘fair’ or ‘poor’) physical and mental health were more likely to report food insecurity (*p* < 0.001, *p* = 0.010), poor sleep quality (*p* = 0.012, *p* < 0.001), and elevated stress (*p* = 0.001, *p* < 0.001). In addition, fast-food consumption (*p* < 0.001), breakfast consumption (*p* = 0.031), and food insecurity (*p* = 0.004) showed changes over three years. **Conclusions**: These findings call for targeted wellness initiatives addressing nutrition, food insecurity, stress management, sleep improvement, and physical activity among students and in university health programs. The FRESH Study emphasizes the need for continued longitudinal research to track health behaviors and inform future interventions.

## 1. Introduction

The undergraduate years are critical for transitioning from adolescence to young adulthood, where students may develop new health behaviors that shape their physical and mental well-being. Especially following the COVID-19 pandemic, the current generation of students has been significantly affected by school closures, remote learning, and limited social connectivity. Urban universities observed deterioration in students’ mental health and disruptions in their daily physical activity, screen time, and sleep quality [1]. The shift in daily routines, social isolation, and increased stress from the pandemic may have altered students’ attitudes toward their health, creating a gap between how they perceive their well-being and their actual health behaviors. Between 2013 and 2023, indicators of poor mental health have worsened among high school students reporting sadness, hopelessness, or suicidality [2]. This trend is particularly concerning for first-year undergraduate students. A previous study found that the transition from high school to university often heightens stress levels and introduces difficulties that intensify these mental health challenges [3]. Further amplifying changes in physical health, the percentage of overweight or obese undergraduate students rose from 34.2% to 42% between fall 2021 and spring 2024, according to the U.S. National College Health Assessment [4,5]. Between 2021 and 2024, the prevalence of food insecurity has also increased from 38% to 49% of undergraduate students [4,5]. The pandemic further worsened the diet quality of adolescents and young adults, evidenced by a drop in the intake of fruits and vegetables as well as daily breakfast consumption [6]. First-year students are especially vulnerable to these poor health outcomes due to a shift in daily routines, changes in academic workload, social challenges, and newfound self-sufficiency [3].

Given the role of undergraduate years in shaping long-term health behaviors, it is essential to explore how students’ current perceptions of their health align with actual health behaviors. Self-rated health has been recognized as a reliable and efficient tool to monitor health changes and assessment [7,8], yet perceived health may potentially not reflect actual behaviors (e.g., diet, physical activity, etc.) [9]. Studies show the important impact that diet, exercise, stress, and sleep can have on physical and mental health [10,11,12,13,14]. Furthermore, understanding how student health perceptions and measures are changing over the years is key to providing insights into the long-term effects of the pandemic on this generation of young adults. Therefore, the primary goal of this article was to examine the relationship between physical and mental health perceptions with measured health metrics among three first-year cohorts at an urban university.

## 2. Materials and Methods

### 2.1. Study Design

The Fitness, Rest, and Exercise for Strength and Health (FRESH) Study was developed to assess first-year college students’ health behaviors and key health metrics and measure them annually throughout their typical four-year undergraduate experience. The study was established as a sequential cohort design to allow for tracking both individual changes over time and trends across different cohorts. It was created during the COVID-19 lockdown in the spring of 2021 and launched for the return to an in-person campus in the fall of 2021. This timing ensured all data collection reflected a typical college environment. The study protocol includes annual surveys administered to study participants, beginning in their first year at the university. These annual surveys account for changing circumstances, allowing researchers to contextualize and adjust for pandemic-related influences. The surveys include diet, food insecurity, physical activity, sleep, and stress assessments. The FRESH Study protocol has been approved by the George Washington University Institutional Review Board (GWU IRB#NCR213655).

### 2.2. Study Population

The study population consisted of full-time, first-year students enrolled at George Washington University (GW) during the academic years 2021–2022, 2022–2023, and 2023–2024. This urban campus is located in Washington, D.C., enrolling over 11,000 undergraduate students (45.8% White, 12.3% Asian, 10.2% Hispanic or Latino, 10.1% Black or African American, 3.92% Two or More Races, 0.15% American Indian or Alaska Native, and 0.123% Native Hawaiian or Other Pacific Islanders) [15]. First-year students are required to live on campus. For the first year of the study, students depended on cooking in community housing kitchens and eating at nearby restaurants. They paid through a student account balance provided as part of their room-and-board package. During the following year, in the spring of 2023, GW introduced traditional ‘all-you-care-to-eat’ dining halls and limited the number of partnerships subsidizing local restaurants for students. Starting fall 2023, first-year students were strictly required to have a dining plan, representing the final cohort of participants. The school has one on-campus recreation center with exercise equipment, fitness group classes, personal training, and opportunities to join club and intramural sports teams.

### 2.3. Participant Enrollment

Each fall semester, first-year students were recruited in the FRESH Study. Recruitment efforts included reaching out to individual student organizations, undergraduate advisors, the athletic department, housing community coordinators, the campus recreation department, the student life department, and professors from all GW schools. Posted flyers explained the study, including both a link and QR code to an interest form. This form further detailed the study and requested a school email for future communication, including informed consent and survey documents.

Upon the commencement of enrollment efforts, students provided informed consent and completed the survey via an email link through Research Electronic Data Capture (REDCap) software version 13.7.30. The questionnaires were made available on the first day of February and closed in the middle of March. All participants received $15 on their student identification card for completion of the study.

### 2.4. Measures

The survey included the following sections: Demographics, General Health, Sleep Quality Index, Perceived Stress Scale, Physical Activity Questionnaire, Food Questionnaire, and Beverage Questionnaire.

#### 2.4.1. Demographics

Participants were asked for their date of birth, sex assigned at birth, academic year, academic school, race, ethnicity, international student status, living situation, primary language, height, and weight. To understand their current living situation, they were asked about the number of people they live with and whether they live on campus. Participants’ household size can impact health behaviors through shared meal preparation, social support, and stress levels. Their housing location can affect access to resources like dining facilities and recreational spaces, which may further influence students’ health behaviors. Additionally, body mass index (BMI) was calculated using self-reported height and weight. Then, values were categorized using current BMI classification labels [16], initially adopted by the World Health Organization [17]. The BMI classification categories and values include underweight (<18.5), healthy weight (18.5 to <25.0), overweight (25.0 to <30.0), and obese (30.0+). In this study, suboptimal BMI was labeled as not having a ‘healthy weight’ status, which is composed of underweight, overweight, and obese categories.

#### 2.4.2. General Health

This questionnaire was adapted from the 2020 Coronavirus Health and Impact Survey (CRISIS), which has been validated for measuring health perceptions across diverse populations [18]. Participants were asked to rate their overall physical and mental/emotional health on a five-point scale of poor, fair, good, very good, and excellent. These two measures provided data on how students perceived their general health. Additional questions were asked about their COVID-19 health history.

#### 2.4.3. Perceived Stress

Ten questions were drawn from the Perceived Stress Scale (PSS-10) [19]. PSS-10 is a psychological tool to measure how individuals perceive their life circumstances within the last month as stressful. This widely used instrument was kept in its original format, ensuring the maintenance of its reliability and validity. All questions were ranked on a five-point scale of ‘never’, ‘almost never’, ‘sometimes’, ‘fairly often’, and ‘very often’. The stress metric used for data analysis was based on the question: “In the last month, how often have you felt difficulties were piling up so high that you could not overcome them?” [19]. Those who declared ‘fairly often’ or ‘very often’ were classified as having a high level of stress accumulation.

#### 2.4.4. Sleep Quality

This questionnaire was implemented from the Pittsburgh Sleep Quality Index Questionnaire (PSQI), which is known for its high internal consistency, test–retest reliability, and strong construct validity [20]. It was utilized to assess usual sleep patterns, quality, and disturbances during the past month. Questions specifically inquired about bedtime, time taken to fall asleep, wake-up time, total hours of sleep, and overall sleep quality. The measure of overall sleep quality was utilized for data analysis and determined by a rating of ‘very bad’, ‘fairly bad’, ‘fairly good’, and ‘very good’. Those who rated their sleep quality as ‘very bad’ or ‘fairly bad’ were indicated as having poor sleep quality.

#### 2.4.5. Physical Activity

The questions on physical activity were designed from the International Physical Activity Questionnaire (IPAQ), which has been thoroughly tested across several countries, showing acceptable criterion validity and good test–retest correlations [21]. Questions investigated time spent doing vigorous physical activity, moderate physical activity, and sedentary activities within the past seven days. In addition, they probed the usage of television, social media, and video games. Moderate and vigorous activity were averaged, calculated into minutes per week, and compared to 150 min per week as recommended by the Physical Activity Guidelines for Americans [22].

#### 2.4.6. Dietary Intake

The NHANES Food Frequency Questionnaire is proven to effectively represent dietary habits across diverse U.S. populations [23]. In this study, diet-related questions were adapted from this tool, investigating the frequency of food and drink choices over the past week. The survey included questions on breakfast consumption, general dietary patterns, fruit and vegetable intake, fast-food consumption, and financial stress regarding food. Beverage-related questions inquired how often the following beverages were consumed: water, fruit juice, sweetened juice, vegetable juices, milk, milk alternatives, soft drinks, diet soft drinks, tea, coffee, protein shakes, sports drinks, and energy drinks [23]. The sugar-sweetened beverages (SSBs) used for data analysis included consumption of sweetened teas, juices, soft drinks, sports drinks, and energy drinks. Drinking four or more SSBs per week was considered high SSB consumption. The two questions regarding fast food and breakfast consumption included a list of frequencies (e.g., never or rarely, 1 time per week, 2 to 3 times per week). High fast-food consumption was indicated by more than 1 time per week, and low breakfast consumption consisted of less than 5 days per week. Lastly, screening for food insecurity was determined by a screening question from the U.S. Adult Food Security Survey [24] in which participants stated ‘yes’, ‘no’, or ‘prefer not to answer’.

### 2.5. Statistical Analysis

Descriptive statistics (frequencies, means, and standard deviations) on salient variables were performed on R version 4.3.3 (R Core Team, 2023) and Stata 18 BE (College Station, TX, USA). Perceived mental and physical health variables were dichotomized for analysis and frequency data. Those who reported ‘excellent’, ‘very good’, or ‘good’ mental and physical health were categorized as optimal. Those who reported ‘fair’ or ‘poor’ mental and physical health were in the suboptimal category. Additionally, key suboptimal health metrics were presented as frequencies and percentages for high stress accumulation, poor sleep quality, low physical activity, high fast-food consumption, high SSB consumption, low breakfast consumption, the prevalence of food insecurity, and not ‘healthy weight’ BMI status. Physical activity and BMI data with a standard deviation greater than 3.0 were excluded from the analysis. For each suboptimal health metric, chi-square tests were used to assess for a significant difference between the three first-year cohorts. Chi-square tests were also used to determine the association between the suboptimal metrics and students’ perception of their physical or mental health. A *p*-value less than or equal to 0.05 was considered statistically significant.

## 3. Results

### 3.1. Participant Demographics

Table 1 demonstrates the demographic characteristics of the participants (*n* = 271), including age, sex, race, and BMI. Overall, the majority were female (76.4%) with the percentage of male participants almost doubled over the three years from 16.4% to 31.1%. The sample represented all racial groups. Caucasian/White students made up the largest group (56.1%), followed by Multiracial (15.9%), Asian (15.9%), African American/Black (9.6%), and Hispanic/Latino (2.6%) students. Concerning BMI categories, most students (68.9%) across all cohorts were classified as having a “healthy weight.” However, the percentage of students classified as “overweight” (26.7%) and “obese” (6.7%) was highest in the third academic year, 2023–2024.

### 3.2. Health Metrics Across the Cohorts

Table 2 presents students’ reported behaviors from each cohort. Of note, three dietary intake metrics showed statistically significant changes across the three cohort years. Fast-food consumption drastically declined from 86.7% to 37.6%, and then to 27.4% over the three consecutive years (*p* < 0.001). On average, for the total study population, more than half (56.5%) of students consumed breakfast daily, and students in the second cohort (2022–2023) ate breakfast less frequently compared to the other two cohorts (*p* = 0.031). In addition, food insecurity prevalence was also the highest during the second-year cohort at 35.8% (*p* = 0.004) and dropped to 15.5% the following year (2023–2024). Almost half (42.2%) of students consumed some type of sugar-sweetened beverage (SSB) more than three times per week on average, yet there were no significant changes between the three years.

Table 2 also reveals the physical and mental health perceptions over the three study waves. In the 2021–2022 cohort immediately following the pandemic period, 55.2% of students rated their mental health as ‘fair’ or ‘poor’, compared to 37.7% in 2022–2023 and 24.7% in 2023–2024, with this improvement in mental health over the years being statistically significant (*p* = 0.001). The most prevalent rating of mental health was ‘fair’ in the first cohort (41.8%; *n* = 67), ‘good’ in the second cohort (37.7%; *n* = 114), and ‘very good’ in the third cohort (40.4%; *n* = 89). The ratings of physical health remained relatively consistent across the three years, with the most prevalent categories being ‘very good’ (40.4%; *n* = 270) and ‘good’ (35.9%; *n* = 270) for the total population. Overall, a large majority (89.6%) of participants rated their physical health as ‘excellent’, ‘very good’, or ‘good’ across the three cohorts. However, calculations from self-reported health measures showed that 34.5% of students had suboptimal levels of physical activity engagement (not meeting the recommended 150 min of moderate-to-vigorous physical activity per week). Thirty-three individuals (13.1% of the 252 who reported their physical activity) stated performing no moderate or vigorous exercise in the last week. Furthermore, 30.7% of the total study population did not have a ‘healthy weight’ status based on BMI classifications.

Neither reported stress nor sleep levels changed significantly over the three study waves. Regarding stress, most participants (71.2%) felt “nervous or stressed” ‘fairly often’ or ‘very often’. Zero students surveyed ‘never’ feeling stressed in the first two cohorts (*n* = 56; *n* = 112), yet seven in the last cohort (2023–2024) surveyed ‘never’ feeling stressed (*n* = 89). For the first studied year, 85.7% (*n* = 56) of students indicated that their stress levels increased, whether ‘a little’ or ‘a lot’, since their arrival to college. This consensus was also persistent with the majority for the 2022–2023 (73.2%) and 2023–2024 cohorts (64.0%). More than a quarter of total students (26.4%) reported poor sleep quality. In the first cohort, the average hours of sleep per night was 7.4, which fell to 7.1 h per night in the last two cohorts.

### 3.3. Perceived Physical Health vs. Measured Health Metrics

Associations between the students’ self-reported physical health and relevant health behaviors are reported in Table 3. Overall, students who reported suboptimal (‘fair’ or ‘poor’) physical health were significantly more likely to report ‘fairly bad’ or ‘very bad’ sleep quality (*p* = 0.012) compared to those who described their perceived physical health as optimal (‘good’, ‘very good’, or ‘excellent’). Among students who report suboptimal physical health, a higher proportion (57.7%) experienced “stress difficulties piling up” ‘fairly often’ or ‘very often’, compared to only 25.1% of those stating optimal physical health (*p* = 0.001). Food insecurity was also significantly associated with perceived physical health. Those who indicated suboptimal physical health were more likely to report being food insecure (60.0% vs. 24.3%; *p* < 0.001). The relationship between perceived physical health and BMI (*p* = 0.060) and reported physical activity (*p* = 0.057) was close to reaching statistical significance.

### 3.4. Perceived Mental Health vs. Measured Health Metrics

Associations between the students’ self-reported mental health and key health behaviors are stated in Table 4. Similar to perceived physical health, mental health perceptions had a strong and significant association with stress accumulation (*p* < 0.001), sleep quality (*p* < 0.001), and food insecurity (*p* = 0.010). In addition, participants with ‘fair’ or ‘poor’ mental health were significantly more likely to consume fast food more than once per week (56.4% vs. 39.6%, *p* = 0.014).

## 4. Discussion

The FRESH Study was initiated to understand the health behaviors among college undergraduate students in the post-COVID era, as well as to examine how these behaviors and measures change in the ensuing years. Key findings include that the majority of students rate their physical health favorably, but many, although improving over subsequent years, rate mental health unfavorably. Of note, findings suggest that perceptions of physical health and mental health may not consistently align with measured health behaviors. Among those who rated their physical health favorably, many had suboptimal dietary patterns, and a significant proportion did not meet recommended physical activity guidelines and were classified as having a BMI outside the ‘healthy weight’ range. Perceived mental health improved significantly over the three academic years, yet a high prevalence of stress, poor sleep quality, and food insecurity persisted, which align as close determinants of mental health [25,26,27]. This disconnect signifies that while health perceptions and self-assessments provide valuable insight, they should be interpreted alongside other behavioral measures for a comprehensive understanding of health status.

First-year students’ nutritional habits on campus warrant attention. Over half of students reported not eating breakfast regularly, which is notable as regular breakfast consumers are more likely to follow other healthy dietary behaviors [28]. Breakfast can provide a significant contribution to the intake of micronutrients and fiber to maintain blood sugar levels and favorable metabolic outcomes throughout the day [28,29]. The changes in food options and the piloting of the campus dining hall may have also negatively affected breakfast habits during the second academic year. Furthermore, fast-food consumption was higher during the first study year but dropped in later cohorts. The introduction of the ‘all-you-care-to-eat’ dining hall and fewer university–restaurant partnerships may have led to students’ decrease in fast-food consumption over the three years. College students’ diets rely heavily on their campus layout and the accessibility of food. Additionally, almost half of the total study population revealed a trend of drinking suboptimal amounts of SSB weekly. Further research is needed to identify and address the factors influencing college students to drink sugar-sweetened beverages regularly.

Food insecurity on campus warrants more attention. Not surprisingly, the prevalence of food insecurity was highest in the students who self-reported their physical and mental health as ‘fair’ or ‘poor.’ Among the three cohorts, the students who reported in the spring of 2023 (second cohort) were the most food insecure. Spring of 2023 was also the introductory pilot semester of the campus dining hall. Simultaneously, the university limited the number of partnerships to local restaurants for students. The adjustments of the dining changes during this single semester may be a major factor in this elevated proportion. It was not until the following fall of 2023, when all first-year students were strictly required to have an unlimited meal plan for the dining hall, set up at the beginning of the year. Due to the elimination of constant reminders of paying for food, this may have led to the drop in food insecurity in the third cohort. At the time the first cohort completed this study in March of 2022, the consumer price index was 292.2 in the D.C. metropolitan area [30]. Through the impact of inflation, there was a 20.9% rise to 313.1 in March 2024. Students are at risk due to limited financial resources and rising costs of tuition, housing, and food. The economic strain and fewer dining options likely contributed to the increased food insecurity trend seen from this study. Historically, food insecurity among first-year college students can have an impact on academic performance and health outcomes [27]. In turn, college students can adopt habits of eating cheaply for hunger rather than for adequate nutrients and energy. Even with a fair knowledge of healthy dietary requirements, college students prioritize convenience, cost, time, and taste [31,32]. Potential solutions may include confidential screening and campus resources to provide them with additional food access [27]. Further examination is warranted to better understand how factors, such as the type of food available for different cultures and dietary restrictions, open dining hours, and inflation impact students’ risk for food insecurity.

The mental health of recent generations has become a chief public health issue, and the FRESH Study further supported this concern. While students commonly rated their physical health positively, many students generally reported their mental health as ‘fair’ or ‘poor’. These critical transition years present young adults with life stressors that display a significant negative correlation with their mental health. Especially following the pandemic, these stressors included concerns for academic performance, disruptions in sleeping patterns, decreased social interactions, worry for loved ones, and difficulty concentrating [33]. Increased stress, if not appropriately managed, can hurt students’ mental well-being and lead to detrimental coping mechanisms [33,34,35]. Consistent with similar research findings [25], over one-third of study participants indicated feeling high stress accumulation during the 2021–2022 school year. Our results showed notable improvements in stress accumulation and mental health over the three years. Possible explanations for this trend may be the adaptations to post-COVID-19 life and increased social connectivity opportunities, which enhance peer support. Improved mental health among college students may enhance academic performance and encourage healthier coping mechanisms. It also may reduce the demand on university resources while promoting career readiness and long-term well-being. The observed relationship between stress and mental health could assist in guiding resource allocation and recommendations for undergraduate students. Evidence illustrates that stress management training can significantly promote psychological well-being and academic vitality in college students [36]. Universities could implement peer support programs, academic resources, or counseling services to improve students’ stress levels. Providing tailored interventions for sleep hygiene, physical activity, and social engagement could work synergistically in helping students better manage their stress and overall health.

Physical and mental health perceptions were also highly associated with sleep quality in our population. The average amount of sleep for all three cohorts was at least 7 h, meeting the recommended minimum hours per night for adults [37]; however, there is still a notable prevalence of poor sleep quality in the study population. Sleep quality, commonly indicated by restfulness, has been assessed as a more superior index than sleep quantity [38]. The duration of sleep may not always have a direct correlation with quality sleep, so it is critical to understand both variables when assessing and treating sleep issues in a college population. Harmful to physical health, acute sleep deprivation can negatively affect immune, inflammatory, and endocrine functioning [39]. Simultaneously, it can damage mental health by standardizing negative emotions, such as anxiety, fatigue, and depression [39]. Feasible programs for training sleep problems in university students confirm significant improvements in subjective sleep quality and sleep-related personality traits [26]. Public health professionals must prioritize the need for action-based programs to better sleep habits in college populations.

During their undergraduate experience, college students have the autonomy to form their own exercise habits. It is notable that over one-third of students are still not meeting the weekly recommendations for physical activity. During the 2021–2022 academic year, the on-campus recreation center and intramural sports were either closed or not offered. As on-campus gym spaces reopened and intramural sports resumed during the 2022–2023 academic year, students in this cohort reported higher levels of intentional physical activity. Universities need to emphasize the availability of resources that promote exercise for students. A longitudinal study examined the physical behaviors of college students, finding a significant decline in daily moderate-to-vigorous physical activity over seven semesters, which was exacerbated when students moved off-campus [40]. Especially at urban universities, residency location, campus walkability, and transportation time to sporting areas can also majorly impact physical activity levels [41]. Accessibility of spaces is as equally important as availability. Through convenience, universities can help first-year students build active habits that persist throughout their future lives. In a previous study on college students’ fitness, lower levels of vigorous activity were associated with higher study time and credit load; higher BMI was associated with higher GPA [42]. As students become busier academically throughout their undergraduate experience, prioritization of exercise may be hindered, so it is vital to assist first-year students in building a healthy lifestyle as they establish their independence.

The FRESH Study has its limitations. First, the use of self-reported data may introduce response bias, including overreporting or underreporting health behaviors due to social desirability. Second, the relatively small, varied sample sizes and skewed representation of the student body limit the generalizability of the FRESH Study to other college populations. The most common demographic group at GW is a white female [15], yet this race and sex were still overrepresented in this study. Given that participation in this study is voluntary, non-response bias is also a concern, potentially skewing the results toward more health-conscious individuals. Recruitment efforts should continue to focus on increasing the diversity of the study population to better reflect the overall college student community. However, notable strengths include the diversity of health metrics, consistent implementation of the survey across several study years and recruitment of new students, and real-time relevancy of data collection to inform university-wide programs. Finally, analyzing longitudinal data as the FRESH Study progresses will explain temporal relationships and promote evidence-based interventions for student health. In controlling for potential confounders, future research should explore how sociodemographic factors shape health behaviors and perceptions.

## 5. Conclusions

The FRESH Study emphasizes the persistent health challenges faced by first-year college students. High sugar-sweetened beverage consumption, low breakfast intake, food insecurity, suboptimal physical activity levels, poor sleep quality, and stress accumulation remain substantial issues among this population. These behaviors, particularly in relation to students’ mental and physical health, underscore the importance of targeted wellness initiatives in university settings. Universities must prioritize stress management programs, nutrition and fitness campaigns, sleep hygiene education, and food insecurity resources. This study calls for a multifaceted approach to student well-being to encourage long-term support. The FRESH Study aims to continue its longitudinal approach in future research, exploring how health behaviors evolve throughout students’ undergraduate years and identifying key interventions to support their overall well-being.

## Figures and Tables

**Table 1:** Demographic characteristics of FRESH study participants by cohort, *n* (%).

Characteristics	2021–2022 Cohort (*n* = 67)	2022–2023 Cohort (*n* = 114)	2023–2024 Cohort (*n* = 90)	Total (*n* = 271)
**Age** (mean ± SD; *n* = 268)	18.4 ± 0.5	18.4 ± 0.6	18.5 ± 0.6	18.4 ± 0.6
**Sex**				
Female	56 (83.6)	89 (78.1)	62 (68.9)	207 (76.4)
Male	11 (16.4)	25 (21.9)	28 (31.1)	64 (23.6)
**Race**				
Hispanic/Latino	2 (3.0)	1 (0.9)	4 (4.4)	7 (2.6)
African American/Black	6 (9.0)	15 (13.2)	5 (5.6)	26 (9.6)
Caucasian/White	37 (55.2)	62 (54.4)	53 (58.9)	152 (56.1)
Asian	13 (19.4)	18 (15.8)	12 (13.3)	43 (15.9)
Multiracial	9 (13.4)	18 (15.8)	16 (17.8)	43 (15.9)
**BMI** (*n* = 270)				
Underweight (<18.5)	5 (7.5)	4 (3.5)	4 (4.4)	13 (4.8)
Healthy Weight (18.5 to <25.0)	45 (67.2)	85 (75.2)	56 (62.2)	186 (68.9)
Overweight (25.0 to <30.0)	13 (19.4)	20 (17.7)	24 (26.7)	57 (21.1)
Obese (30+)	4 (6.0)	4 (3.5)	6 (6.7)	14 (5.2)

**Table 2:** Self-reported health measures of FRESH study participants by cohort, *n* (%).

Health Metrics	2021–2022 Cohort (*n* = 67)	2022–2023 Cohort (*n* = 114)	2023–2024 Cohort (*n* = 90)	*p*-Value	Total (*n* = 271)
**Overall Physical Health** (*n* = 270)					
Good, Very Good, or Excellent	59 (88.1)	102 (89.5)	81 (91.0)	0.834	242 (89.6)
Fair or Poor	8 (11.9)	12 (10.5)	8 (9.0)		28 (10.4)
**Overall Mental Health** (*n* = 270)					
Good, Very Good, or Excellent	30 (44.8)	71 (62.3)	67 (75.3)	0.001 *	168 (62.2)
Fair or Poor	37 (55.2)	43 (37.7)	22 (24.7)		102 (37.8)
**Not ‘Healthy Weight’ Status** ^1^ (BMI; *n* = 270)	22 (32.8)	28 (24.8)	33 (36.7)	0.173	83 (30.7)
**Low Physical Activity** **<150 min/week** (*n* = 252)	26 (44.1)	32 (29.6)	29 (34.1)	0.171	87 (34.5)
**High Stress Accumulation** (*n* = 257)	21 (37.5)	30 (26.8)	22 (24.7)	0.221	72 (28.4)
**Poor Sleep Quality** (*n* = 254)	17 (29.8)	32 (28.8)	18 (20.9)	0.367	67 (26.4)
**High SSB Consumption** ^2^ **>3 times/week** (*n* = 258)	22 (34.4)	46 (42.2)	41 (48.2)	0.238	109 (42.2)
**High Fast-Food Consumption > 1/week** (*n* = 253)	52 (86.7)	41 (37.6)	23 (27.4)	<0.001 *	116 (45.8)
**Low Breakfast Consumption** **<5 days/week** (*n* = 260)	34 (50.7)	72 (66.1)	41 (48.8)	0.031 *	147 (56.5)
**Food Insecurity Prevalence** (*n* = 260)	20 (29.9)	39 (35.8)	13 (15.5)	0.004 *	72 (27.7)

^1^ Not ‘healthy weight’ includes underweight, overweight, and obese BMI categories. ^2^ Sugar-sweetened beverage (SSB). * *p*-value ≤ 0.05.

**Table 3:** Relationship between students’ perceived physical health and suboptimal health indices, *n* (%).

Health Metric	Level	Perceived Physical Health (*n* = 270)		*p*-Value
		Fair or Poor (*n* = 102)	Good, Very Good, or Excellent (*n* = 168)	
**BMI** (*n* = 269)	‘Healthy Weight’ Status	14 (51.9)	173 (71.5)	0.060
	Not ‘Healthy Weight’ Status ^1^	13 (48.1)	69 (28.5)	
**Physical Activity** (*n* = 252)	≥150 min/week	11 (45.8)	154 (67.5)	0.057
	<150 min/week	13 (54.2)	74 (32.5)	
**Stress Accumulation** (*n* = 257)	Never, almost never, sometimes	11 (42.3)	173 (74.9)	0.001 *
	Fairly often, very often	15 (57.7)	58 (25.1)	
**Sleep Quality** (*n* = 254)	Very good, fairly good	12 (50.0)	175 (76.1)	0.012 *
	Fairly bad, very bad	12 (50.0)	55 (23.9)	
**SSB Consumption** ^2^ (*n* = 258)	≤3 drinks/week	14 (56.0)	135 (57.9)	1.000
	>3 drinks/week	11 (44.0)	98 (42.1)	
**Fast-Food Consumption** (*n* = 253)	≤1 time/week	10 (40.0)	127 (55.7)	0.199
	>1 time/week	15 (60.0)	101 (44.3)	
**Breakfast Consumption** (*n* = 260)	≥5 days/week	8 (32.0)	105 (44.7)	0.315
	<5 days/week	17 (68.0)	130 (55.3)	
**Food Insecurity** (*n* = 260)	No	9 (36.0)	173 (73.6)	<0.001 *
	Yes	15 (60.0)	57 (24.3)	
	Prefer not to answer	1 (4.0)	5 (2.1)	

^1^ Not ‘healthy weight’ includes underweight, overweight, and obese BMI categories. ^2^ Sugar-sweetened beverage (SSB). * *p*-value ≤ 0.05.

**Table 4:** Relationship between students’ perceived mental health and suboptimal health indices, *n* (%).

Health Metric	Level	Perceived Mental Health (*n* = 270)		*p*-Value
		Fair or Poor (*n* = 102)	Good, Very Good, or Excellent (*n* = 168)	
**BMI** (*n* = 269)	‘Healthy Weight’ Status	69 (68.3)	118 (70.2)	0.846
	Not ‘Healthy Weight’ Status ^1^	32 (31.7)	50 (29.8)	
**Physical Activity** (*n* = 252)	≥150 min/week	55 (60.4)	110 (68.3)	0.260
	<150 min/week	36 (39.6)	51 (31.7)	
**Stress Accumulation** (*n* = 257)	Never, almost never, sometimes	39 (41.9)	145 (88.4)	<0.001 *
	Fairly often, very often	54 (58.1)	19 (11.6)	
**Sleep Quality** (*n* = 254)	Very good, fairly good	48 (52.7)	139 (85.3)	<0.001 *
	Fairly bad, very bad	43 (47.3)	24 (14.7)	
**SSB Consumption** ^2^ (*n* = 258)	≤3 drinks/week	58 (61.1)	91 (55.8)	0.491
	>3 drinks/week	37 (38.9)	72 (44.2)	
**Fast-Food Consumption** (*n* = 253)	≤1 time/week	41 (43.6)	96 (60.4)	0.014 *
	>1 time/week	53 (56.4)	63 (39.6)	
**Breakfast Consumption** (*n* = 260)	≥5 days/week	37 (38.1)	76 (46.6)	0.228
	<5 days/week	60 (61.9)	87 (53.4)	
**Food Insecurity** (*n* = 260)	No	57 (58.8)	125 (76.7)	0.010 *
	Yes	37 (38.1)	35 (21.5)	
	Prefer not to answer	3 (3.1)	3 (1.8)	

^1^ Not ‘healthy weight’ includes underweight, overweight, and obese BMI categories. ^2^ Sugar-sweetened beverage (SSB). * *p*-value ≤ 0.05.

## Data Availability

The dataset presented in this article is not readily available because the data are part of an ongoing study. Requests to access the datasets should be directed to Jennifer Sacheck.
